# Role of Artificial Intelligence in Global Surgery: A Review of Opportunities and Challenges

**DOI:** 10.7759/cureus.43192

**Published:** 2023-08-09

**Authors:** Kashish Malhotra, Benjamin Ngie Xiong Wong, Susie Lee, Helena Franco, Carol Singh, Laura A Cabrera Silva, Habab Iraqi, Akatya Sinha, Sule Burger, Danyca Shadé Breedt, Kashish Goyal, Mert Marcel Dagli, Ashvind Bawa

**Affiliations:** 1 Department of Surgery, Dayanand Medical College and Hospital, Ludhiana, IND; 2 Department of Surgery, Austin Hospital, Melbourne, AUS; 3 Department of Orthopaedics, Toowoomba Hospital, Queensland, AUS; 4 Department of Surgery, Bond University, Queensland, AUS; 5 Department of Surgery, Universidad El Bosque, Bogotá, COL; 6 Department of Surgery, Al-Yarmouk College of Medical Sciences, Khartoum, SDN; 7 Department of Surgery, MGM (Mahatma Gandhi Mission’s) Medical College and Hospital, Mumbai, IND; 8 Department of Surgery, Ngwelezana Hospital, KwaZulu-Natal, ZAF; 9 Department of Surgery, King Edward VIII Hospital, Durban, ZAF; 10 Department of Internal Medicine, Dayanand Medical College and Hospital, Ludhiana, IND; 11 Department of Neurosurgery, University of Pennsylvania Perelman School of Medicine, Philadelphia, USA

**Keywords:** low- and-middle-income countries, ai and robotics in healthcare, surgical equity, global health, global surgery, artificial intelligence

## Abstract

Global surgery broadly refers to a rapidly expanding multidisciplinary field concerned with providing better and equitable surgical care across international health systems. Global surgery initiatives primarily focus on capacity building, advocacy, education, research, and policy development in low- and middle-income countries (LMICs). The inadequate surgical, anesthetic, and obstetric care currently contributes to 18 million preventable deaths each year. Hence, there is a growing interest in the rapid growth of artificial intelligence (AI) that provides a distinctive opportunity to enhance surgical services in LMICs. AI modalities have been used for personalizing surgical education, automating administrative tasks, and developing realistic and cost-effective simulation-training programs with provisions for people with special needs. Furthermore, AI may assist with providing insights for governance, infrastructure development, and monitoring/predicting stock take or logistics failure that can help in strengthening global surgery pillars. Numerous AI-assisted telemedicine-based platforms have allowed healthcare professionals to virtually assist in complex surgeries that may help to improve surgical accessibility across LMICs. Challenges in implementing AI technology include the misrepresentation of minority populations in the datasets leading to discriminatory bias. Human hesitancy, employment uncertainty, automation bias, and role of confounding factors need to be further studied for equitable utilization of AI. With a focused and evidence-based approach, AI could help several LMICs overcome bureaucratic inefficiency and develop more efficient surgical systems.

## Introduction and background

The exponential growth of computing power in the 21st century has led to the application of artificial intelligence (AI) in the field of healthcare. AI is used as an umbrella term for the scientific discipline that focuses on the wide range of algorithmic programs and models to perform numerous complex tasks constituting the application of mathematics, statistics, and computer sciences to mimic the simulation of intelligent behavior [1-3].

Global surgery broadly refers to a rapidly expanding multidisciplinary field concerned with providing better and equitable surgical care across international health systems. Global surgery initiatives primarily focus on capacity building, advocacy, education, research, and policy development in low- and middle-income countries (LMICs) [4-6]. In 2015, the Lancet Commission on Global Surgery identified inadequate surgical, anesthetic, and obstetric care as a contributing factor to 18 million preventable deaths each year [7].

Thereafter, The Lancet and the Financial Times launched a collaborative commission focused on the confluence of digital health, AI, and universal health coverage in October 2019 [8]. The rapid growth of AI technology provides a unique opportunity to investigate possible benefits in the provision of health services delivery to LMICs around the world [9]. Although AI has been used in numerous ways in high-income countries, its application still remains emerging but nascent in less resourceful countries [3]. Furthermore, various studies found that doctors and medical students had limited knowledge of the utility, principles, limitations, and ethical usage of AI [10,11].

It is also imperative to acknowledge the disparities of global health coverage particularly in the provision of surgical procedures between developed regions and LMICs. A recently published study found that there was significant gender inequity among global surgery researchers with limited involvement from low- and middle-income countries [12]. In the context of these efforts to emphasize global surgery and ensure universal health coverage, we aim to identify the opportunities and challenges applicable to the usage of AI in global surgery. We discuss the types of AI with applications in surgery in a focused way and reflect on the opportunities available for the application of AI in global surgery in various interconnected disciplines. We also discuss the various challenges that must be addressed before AI can be fully utilized to tackle surgical inequities in under-resourced population as it is critical that the adoption of AI in low- and middle-income countries must occur through an inclusive and sustainable process.

## Review

Types of AI and its applications

There is a wide spectrum of AI technologies that are being used in healthcare services with broad predominant techniques being machine learning (ML), artificial neural networks (ANNs), deep learning (DL), computer vision (CV), natural language processing (NLP), reinforcement learning (RL), fuzzy logics, robotics, and cybernetics. Through the use of algorithms and pattern recognition, ML can analyze real-world data and estimate an outcome [13,14]. The machine’s algorithm (supervised or unsupervised) can become more accurate and predictive with repetition and more input as it can identify and make inferences between datasets [13]. This potential has enabled ML to be utilized in making diagnoses based on imaging, assessing disease severity, and contributing to health policy and planning [9].

ANN is an advanced subset of ML [15]. Similar to the human brain, networks of interconnected computer processors called "neurons" can process data inputs and generate an output [13]. Each node can accept input and store some information about it before passing the data to the next level. This results in each layer having an increasingly complex understanding of the information than the previous layer [16].

Multiple layers of interconnected neurons have led to the development of DL [17]. This subset of ML comprised numerous complex algorithms that can identify the key features of a model without human input [14]. Examples of deep learning include speech recognition and object detection [18]. It has been proposed that DL could improve the objectivity of diagnosis in the field of pathology to assist healthcare professionals to make a more rapid diagnosis and treatment plan. This has been shown in the diagnosis of prostate cancer and the detection of lymph node metastasis in patients with breast cancer [19].

The applications of these subsets of AI have further led to the development of advanced technologies. Intraoperative video can be analyzed with CV which can help in the reliable identification of surgical phases (steps) and tools across a range of operations. CV can even detect operative phases with similar accuracy as surgeons in some circumstances [20]. The use of NLP to extract surgical outcomes from electronic health records, particularly postoperative problems, is growing across disciplines, and NLP may be superior in ruling out documentation of surgical outcomes than traditional non-NLP approaches in some aspects [21].

Reinforcement learning has the potential to assist in surgical decision-making by recommending actions at predefined intervals which mimics a human trial-and-error learning process to calculate optimum recommendation policies [22]. Fuzzy logic inference has been used as a sensitive method of predicting patients who will fail to improve with surgical intervention which employs the principles of contingent probability and involves the recognition of intermediate logical values [23]. Cybernetics and robotic surgeries are additional transdisciplinary domains that may help to augment and enhance the ability to do some surgical procedures safely [24]. Table 1 compiles the broad types and applications of AI commonly used in surgery.

**Table 1:** Broad types and applications of artificial intelligence in surgery.

Types of artificial intelligence (AI)	Working mechanism	Common applications in surgery
Machine learning	Broad subset of AI aimed at automating analytical modeling and improving the findings without being explicitly programmed to do so.	To calculate predictions, accuracy levels, and analysis of various outcomes and interventions.
Artificial neural networks	Mimics “biological neuronal synapse” by interconnected computer processors to process information.	Subset of machine learning.
Reinforcement learning	Employs “trial and error” to come up with a solution in order to maximize the notion of cumulative reward.	Subset of machine learning.
Deep learning	Mimics “human brain” to progressively extract higher-level features from the raw input with no or minimal human supervision.	Subset of machine learning.
Natural language processing	Give machines the ability to help understand, interpret, and manipulate human language to extract information and insights contained in the data.	Automates manual assessment process, especially in the records system and to spot quality lapses.
Computer vision	Mimics “human visual system” to derive meaningful information from digital images, videos, and other visual inputs.	Acquisition and interpretation of visual images/videos, leading to image-guided and virtual surgery.
Fuzzy logics	Associates degrees of likely possibilities instead of a hard yes-no type decision to handle vagueness and imprecise information.	Assist decision-making and performance assessment of healthcare providers.
Robotics and cybernetics	Transdisciplinary approach to build automatic control systems.	Training, navigating, and assisting healthcare professionals with a minimally invasive approach.

Pillars of global surgery and AI: opportunities

Education and Training

The shortage of doctors in LMICs has been a long-standing challenge for several key reasons such as poor working conditions, brain drain, insufficient training opportunities, and uneven distribution of resources. For instance, India, the world's most populous nation, faces a nearly 80% shortage of surgeons, gynecologists, and other specialist doctors in rural areas [25]. With such a profound imbalance in supply and demand, the quality of healthcare in LMICs will continue to deteriorate if nothing is done promptly to increase global access to surgical care. AI can greatly help in the training of upcoming healthcare professionals by personalizing education, automating administrative tasks, and hence reducing the workload on teachers and students. For instance, large language learning models based on generative pre-trained transformer have the potential to be incorporated as virtual assistants and streamline various tasks [26]. AI-enhanced Education Management Information System (EMIS) in LMICs further brings an opportunity for more efficient management and administration of an educational system, development of realistic and cost-effective programs, formulation of responsive policies, as well as analyzing and monitoring educational results [27].

Furthermore, AI is a useful aid for people with special needs and helps them train accordingly taking into account their disabilities, therefore, providing individualized learning [28]. AI also helps to identify early warning signs of dropouts and the unmet needs of students. Big data analytics approach has been used in Uruguay to anticipate rising absences and identify students with a potential risk of dropout [29]. Similar systems to promote and monitor surgical education can be implemented which may aid in improving the mental health of upcoming surgeons and improve surgical skills. Algorithms have been developed which can identify the procedure being done and could be useful for complicated surgical procedures or for situations where an inexperienced healthcare professional is required to perform an emergency surgery [30].

The usage of simulation training allows for cost-effective surgical training and a potential avenue for training non-surgical healthcare workers to provide basic surgical care. In a comparative study done in low- and high-resource educational centers, simulation was shown to successfully enhance surgical training in low-resource settings [31]. AI-assisted tools have been used by other frontline workers in guiding them to triage and provide symptom-based care as is currently done in some LMICs [32]. Various types of ANN models have been used to effectively triage patients with high accuracy, clinical decision support, and complication prediction [33]. However, the cost-effectiveness and large-scale application of AI-assisted algorithms and augmented reality software to assist in training in a low-resource setting need to be studied further [34]. Current international simulation-based teaching models such as SIMBA Simulation (Simulation via Instant Messaging-Birmingham Advance) use medical students and junior doctors as moderators to simulate real-life clinical scenarios than AI-assisted teaching [35].

Governance and Infrastructure Development

Overall, surgical interventions are among the most cost-effective procedures in global health. For example, the treatment of congenital surgical conditions costs approximately 12 to 59 United States Dollars (USD) per disability-adjusted life years averted (DALY), in comparison to HIV prevention efforts, which cost between 103 and 302 USD per DALY averted [36-38].

Within each National Surgical, Obstetric, and Anesthesia Plan (NSOAP) domain [5], data drives strategic choices for each country and AI may be used to answer process-related questions and provide insights that aid the policymakers and administrators in advancing surgical systems. AI could help these countries overcome bureaucratic inefficiency and develop more efficient surgical systems by assisting in the production, distribution, and use of the already available health workforce and infrastructure [39].

AI systems that analyze the data transparently may be used in better-allocating system resources and streamlining supply chains [39]. Poor urban planning and inefficient utility distribution are other challenges of poor infrastructure development that can be addressed by AI [40]. This may lead to the development of “smart hospitals” to promote better quality, faster, and sustainable patient-centric care by providing data-driven insights to facilitate decision-making. Setting up AI systems will come at different costs depending on the geographical location that needs to be further studied.

Research and Quality Improvement

Research using AI-augmented technology is becoming increasingly popular in a multitude of different industries including the surgical field. To the researcher, AI assistance will reduce the amount of time spent on a project by automating redundant tasks in a cost-efficient manner, completing tasks more rapidly, reducing errors, and discovering trends and meaning in data more rapidly. Recent advancements include AI-enabled multispectral wound imaging devices that can provide real-time pathogen detection. Numerous projects especially in high-income countries (HICs) have studied AI models in various specialties to improve clinical efficiency and quality improvement with mutual benefits to doctors and patients [41,42]. The establishment of AI-based complaint management systems by involving patients may also be helpful with an appropriate regulatory framework [43].

With a shortage of doctors and inequality between rural and urban health, such AI tools provide already overburdened healthcare systems a unique opportunity to improvise and use these advancements to their benefit in LMIC [44]. Accessibility barriers in low-resource settings can be addressed with low-cost interventions using AI tools following the trails of previous successful endeavors in other specialties such as AI-assisted dengue outbreak tool [45]. This may help in early risk mitigation and management for surgical scenarios more effectively. Additionally, the usage of AI in drug designing and vaccine development may aim at primordial, primary, secondary, and tertiary levels of prevention and help reduce the overall disease burden.

Logistics and Surgical Service Delivery

Systems requiring the importation of components, complex supply chains, or expertise that is not locally supported face significant barriers in providing surgical services. AI has previously been shown to streamline the process of medical equipment delivery and has been a game-changer for developing countries, Zambia and Tanzania, to close the immunization gap [46]. Similar models can be used in procurement and forecasting shortages of surgical equipment with fewer missed opportunities. Machine learning models may also be used to enhance inventory management and supply chains for various other drugs and diagnostic kits, resulting in decreased wastage and optimal inventory.

**Figure 1:**
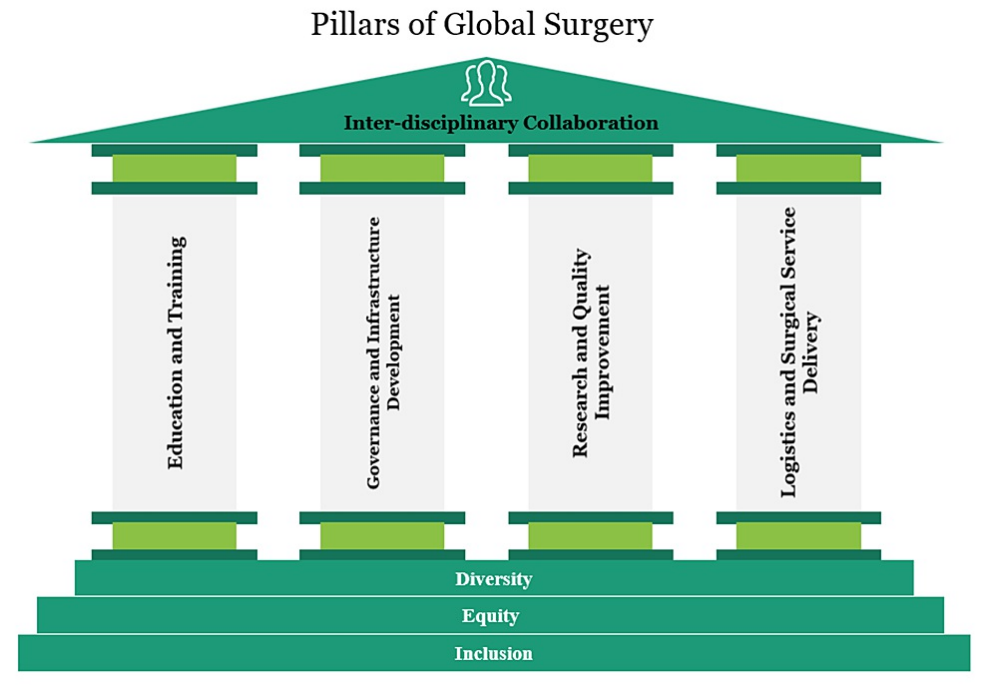
Pillars of global surgery.

Similarly, in the case of local unavailability of superspecialists, AI-assisted tools including telemedicine and augmented reality can be used to interact with a healthcare professional virtually and use their assistance. In Peru and Vietnam, for example, the Proximie app was used to connect local surgical teams with academic centers in the United States and the UK [47]. With this, surgical procedures can be performed more efficiently and safely in LMICs. AI presents an opportunity in the sense that it brings the possibility of having highly trained doctors and nursing staff from other countries virtually to assist in surgery. Figure 1 shows the pillars and foundation stone of global surgery.

Challenges ahead

Representation of Minorities and Ethics

As machines are “trained” by the number of inputs, minority populations will be at inherent risk of underrepresentation as there will be fewer datasets compared to the majority of the population. This may lead to discriminatory bias due to models aimed at representing the majority and not necessarily the minority subgroups [48]. Also, there are different predispositions of various diseases in ethnicities. Separate models running explicitly according to the data obtained from minority subgroups may be implemented. However, if this issue is not properly addressed, it may widen the gap between the quality of care provided to the various horizons of the population. It is crucial to ensure that AI complements and enhances the doctor-patient relationship rather than replacing it [49]. Determining responsibility and liability in cases of AI-related errors or adverse outcomes can be complex. It is essential to establish clear guidelines regarding the usage of AI and ensure that proper informed consent is taken, as some patients may feel uncomfortable with AI involvement in their healthcare decisions. As AI systems often rely on vast amounts of patient data for training and decision-making, ensuring the privacy and security of this data is crucial to prevent unauthorized access or misuse of sensitive health information, as complying with federal regulatory standards.

Financing, Human Hesitancy, and Employment Uncertainty

Rising automation comes at a time when economic inequality is widening, increasing worries of mass technological unemployment and renewing calls for policymakers to address the impact of technological transition. Despite conflicting opinions, there is a possibility that AI may obviate human employment, which naturally causes considerable reluctance to embrace the implementation of AI at the outset even among the medical fraternity who feel unaware and unprepared to incorporate AI in their daily practice [50-52]. In policy development for global surgery, it will be important to focus on achieving a better understanding of human-computer interactions, initial capital requirements, and its implications before making any substantial change in logistics or governance, so as to address the prospect of human hesitancy. Individual policies will be needed to meet the diverse unmet needs of various economies with some facing internal political instability as well. Collective intra- and inter-governmental cooperation will be needed for the holistic success of AI tools and human resource development.

Regulation and Oversight of High-Quality Data

For machine learning models directed at patients, special regulations and framework is needed as a patient may not be able to verify or interpret the results [2]. Automation bias may affect the ability of doctors to provide quality care if there is an overdependence on machine learning models in making decisions [53]. The use of smaller datasets with noisy inputs may lead to suboptimal results needing a clinician’s critical assessment. Asynchronous and asymmetric data from LMICs which may not necessarily be of low quality but varied in type also needs to be handled accordingly [39].

Confounders, Manipulation of Findings, and the Black-Box Problem

Adversarial attacks may also lead to the manipulation of findings. Machine learning models may exploit unknown confounders to achieve the best prediction which may lead to erroneous results later [54]. The advantage of AI to self-learn and update may be at the trade-off of multiple risks including privacy, accuracy, and cybersecurity. The challenge faced with black-box neural networks is their inability to provide justifications and pathways that lead to the final result. Regulators, providers, and researchers need to collaborate to address this issue [55,56].

**Figure 2:**
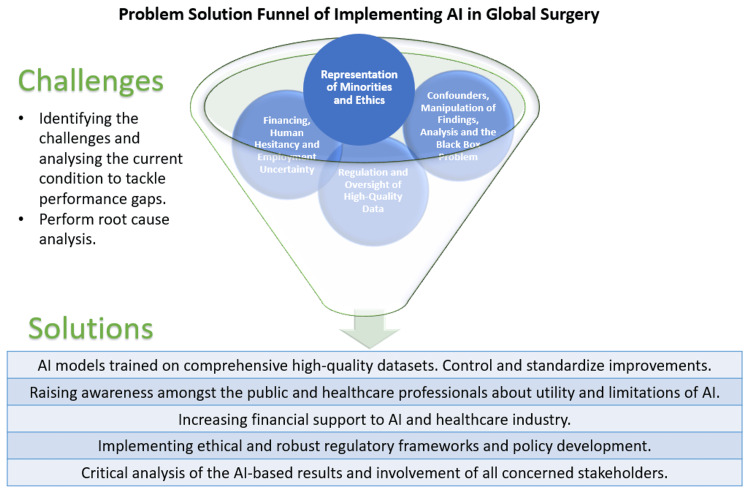
Problem-solution funnel of implementing artificial intelligence in global surgery. AI: artificial intelligence.

In some machine learning models, the emphasis is put to correlate input and output data without a focus on causality. This leads to an inherent flaw that correlation does not imply causation. For these models, it can only be used as an approximation and not as a trusted answer [57]. In addition, metrics do not always represent applicability [54]. Because no one metric can capture all of the desirable qualities of a model, numerous indices are often used to summarize its performance. However, these metrics may not represent the actual clinical applicability and whether their use will bring a positive change in governance or patient care. Figure 2 depicts the problem-solution funnel of the implementation of AI in global surgery.

## Conclusions

Artificial intelligence in surgical care systems is expected to undergo substantial growth post the COVID-19 pandemic. The global healthcare infrastructure has observed that to develop and maintain a sustainable healthcare setup, the utilization of computational technologies such as artificial intelligence becomes crucial. In addition, several research centers and governments have actively participated in the building of robust AI technologies which are assisting healthcare professionals to work efficiently even under a shortage of resources. These factors will eventually drive surgical education and training growth. AI is expanding its footprint in clinical systems ranging from databases to intraoperative video analysis. The unique nature of surgical practice leaves doctors and healthcare stakeholders well-positioned to help usher in the next phase of AI, one focused on generating evidence-based, real-time clinical decision support designed to optimize patient care and workflow.
